# Risk factors for hospital-acquired infections in teaching hospitals of Amhara regional state, Ethiopia: A matched-case control study

**DOI:** 10.1371/journal.pone.0181145

**Published:** 2017-07-18

**Authors:** Walelegn Worku Yallew, Abera Kumie, Feleke Moges Yehuala

**Affiliations:** 1 Institute of Public Health, College of Medicine and Health Sciences, University of Gondar, Gondar, Ethiopia; 2 School of Public Health, College of Health Sciences, Addis Ababa University, Addis Ababa, Ethiopia; 3 Department of Medical Microbiology, College of Medicine and Health Sciences, University of Gondar, Gondar, Ethiopia; St George's University of London, UNITED KINGDOM

## Abstract

**Background:**

Hospital-acquired infection affects hundreds of millions of people worldwide. It is a major global issue for patient safety. Understanding the potential risk factors is important to appreciate the local context. A matched case control study design, which is the first of its kind in the study region, was undertaken to identify risk factors in teaching hospitals of Amhara regional state, Ethiopia.

**Method:**

A matched case control study design matched with age and hospital type was used. The study was conducted in University of Gondar and Felege-Hiwot medical teaching hospital. Cases were patients who fulfilled the criteria based on CDC definition of hospital-acquired infection and controls were patients admitted to the hospital that stayed for more than 48 hours in the ward in the study period, but who did not develop infection. For one case, four controls were selected. Of 545 patients, 109 were cases and 436 were controls. Conditional logistic regression using STATA 13 was used for data analysis.

**Result:**

The median length of stay for cases and controls was 7 and 8 days, respectively. Patients admitted in wards with the presence of medical waste container in the room had 82% less chance of developing hospital-acquired infection (AOR 0.18; 95% CI, 0.03–0.98). The odds of developing hospital-acquired infection among immune deficient patients were 2.34 times higher than their counterparts (95% CI; 1.17–4.69). Patients received antimicrobials, central vascular catheter and surgery since admission had 8.63, 6.91 and 2.35 higher odds of developing hospital-acquired infection, respectively.

**Conclusion:**

Health providers and mangers should consider the provision and availability of healthcare materials and facilities in all of the ward rooms, follow appropriate safe medical procedures for use of external devices on patients, and give attention to the immunocompromised patients for the prevention and control of hospital-acquired infections.

## Introduction

Hospitals are the potential source of the risk of acquiring an infection during the healthcare delivery. Hospital-acquired infections(HAIs) are associated with increased attributable mortality, length of stay in the hospital, and healthcare costs incurred by patients and healthcare facilities[1,2]. Hospital-acquired infections are a growing problem at every level of the healthcare system. World Health Organization (WHO) estimated that it affects hundreds of millions of people worldwide and it is a major global issue for patient safety[3]. The prevalence of hospital acquired infections in two teaching hospitals in Ethiopia was 14.9%[4].

Risk of hospital-acquired infections increased with invasive devices used [5] for example, for treatment and monitoring of patient in Intensive Care Units (ICU)[6]. Incidence of device-associated infections(DAIs) as reported by the International Nosocomial Infection Control Consortium(INICC) is higher than reported by the US National Healthcare Safety Network (NHSN)[7,8]. The DAI patients were 5.3% in China[9],12.2% in Colombia[10] and 13% in Peruvians ICU patients[11]. Evidences showed that the device-associated hospital-acquired infections increased the length of stay in hospital [12–14], and increased mortality [10,11,14]. Researches in developing countries showed that the DAIs in intensive care unit were high[15,16] and incurred extra cost for patients[17–19].

Patients admitted to the hospital wards are susceptible to HAIs. Risk factors for such infections vary between different specific site infections, because hospital environments are complex. Previously conducted researches indicated that, longer hospital stays[20,21], gender[22,23], intravascular catheter[24,25], surgery since admission[22,26], intubation[27], mechanical ventilation[28], age of the patient[4,29], type of hospital [4,30], urinary catheter[22,27] were some of the risk factors for hospital acquired infections.

In Ethiopia, though few researches were conducted on specific surgical site infections[31–33] and hospital-acquired infections[4], none of those used strong epidemiological analytical method, to determine important risk factors. Matched case control study also has its own bias during matching[34]. However as compared to cross-sectional design, matched case control design is stronger for risk factor assessment and minimizes bias[35]. There was no such strong evidence generated in the earlier study settings. Therefore, the aim of this study was to identify risk factors of hospital-acquired infections in teaching hospitals of Amhara regional state, Ethiopia. The findings of the study will be used as an input for policy makers, programmers and health care workers to improve the clinical services.

## Materials and methods

A matched case control study design, matched by age ± 5 years and hospital, was conducted at the University of Gondar and Felege-Hiwot medical teaching hospitals of Amhara regional State, Ethiopia. Amhara region is the second most populous region in the country. Data was collected in two phases: the first being from 16 March to 02 April 2015 and the second from 01 July to 10 July 2015. All inpatients admitted to the two hospital wards were included in the study. Surgical, Gynecology & Obstetrics, Internal medicine, Pediatrics, Ophthalmology and Intensive Care Unit wards were included. Emergency and Recovery departments and wards were excluded.

Case***s*** were patients who developed a hospital-acquired infection based on Centers for Disease Control’s definition, i.e., patients admitted to the hospitals and presented an infectious agent(s) or its toxin(s), and that occurred 48 h or more after admission to the hospital that were neither present nor incubating on admission[36].

Controls were the patients admitted to the hospital and stayed for more than 48 hours in the ward in the study period and did not developed a hospital-acquired infection. The controls were matched based on age +5 years within the same hospital. A case to control ratio of 1:4 was used.

The outcome variable was presence of hospital-acquired infections. Intrinsic and extrinsic factors, such as immunodeficiency, insertion of a urinary catheter, peripheral vascular catheter, mechanical ventilation, availability of hand washing material, and McCabe score were assessed. Hospital-acquired infections were confirmed if the patient had signs and symptoms which met the Centers for Disease Control and Prevention (CDC, Atlanta, GA, USA) definition at the time of the data collection[36]. The sample size was calculated by assuming a 5% type I error, 80% power to detect exposure difference between cases and controls, a 1:4 ratio of cases to controls, and a design effect of 1.5. Accordingly, 109 cases and 436 controls, a total of 545 subjects were required for the study.

The study was conducted after ethical approval of Addis Ababa University College of Health Science Institutional Review Board. Data were collected after written consent with a brief description on the importance of the study to the participants. In addition to consents taken from the parent/guardian, for children aged between 7–18 years written assents also were taken from each study participants. A pretested standardized questionnaire was used to collect data. Moreover, medical record and consultation with the person in charge of the patient were the gold standard for the identification of the cases. Data collectors were trained for three days about the definitions and the study protocol prior to starting the study. Double data entry was conducted to minimize errors occurred during data entry.

Data was entered and validated using EPI-INFO software version 3.5.3 (Atlanta, USA) and STATA 13 for analysis. After matching cases and controls with a unique identifier, bivariate and multivariable conditional logistic regression was employed to identify independent factors associated with HAI. A bivariate analysis was run for each variable. Then those variables which were significant at the bivariate analysis along with variables that were well known predictors of HAI were included in the multivariable analysis. Odds ratios with the corresponding 95% confidence intervals (CI) were estimated and p values were determined. Variables with P < 0.05 in the multivariable conditional logistic regression analysis were considered as significant independent predictors of HAI in this study.

## Results

A total of 545 patients were included in this study. One hundred nine were cases and the remaining 436 were controls. The median age of the cases was 25 years (Interquartile range of 16–35) and, for controls 25 years (Interquartile range of 16–36). Length of stay of patients in the hospital was 8 days with Interquartile range of 4–15 days. The median length of stay for cases and controls was 7 and 8 days respectively (Table 1).

**Table 1:** Background characteristics of cases and controls, in teaching hospitals in Amhara region, Ethiopia, 2015.

Characteristics		Cases n = 109	Controls n = 436	P-Value
**hand washing material available in ward**	**Yes**	45(41.3%)	238(54.6%)	0.001
	No	64(58.7%)	198(45.4%)	
**Sex**	Male	64(58.7%)	201((46.1%)	0.01
	Female	45(41.2%)	235(53.9%)	
**Age categorized**	< = 1 year	9(8.2%)	24(5.5%)	
	1–14 year	14(12.84%)	76(17.43%)	
	15–35 years	59(54.13%	255(51.6%	0.999
	36–55 years	22(20.18%)	90(20.64%)	
	> = 56 years	5(4.6%)	21(4.8%)	
**Ward of admission department**	Medicine	21(19.3%)	118(27.1%)	
	pediatrics	19(17.4%	79(18.1%)	
	surgery	53(48.6%)	141(32.3%)	
	Gynecology	16(14.7%)	93(21.3%)	
	Ophthalmology	0(0%)	5(1.1%)	
**hand rubs available in the ward**	Yes	67(61.5%)	302(69.3%)	0.003
	No	42(38.5%)	134(30.7%)	
**Presence of medical waste container in the ward**	Yes	102(93.6%)	431(98.9%)	0.04
	No	7(6.4%)	5(1.1%)	
**Diabetics History**	Yes	3(2.8%)	9 (2.1%)	
	No	102 (93.6%)	402 (92.2%)	0.400
	Unknown	4(3.7%)	25(5.7%)	
**Immune deficiency**	Yes	31(28.4%)	92(21.1%)	0.018
	No	55(50.5%)	279 (64.0%)	
	Unknown	23(21.1%)	65(14.9%)	
**McCabe score**	Non-Fatal diseases	47 (43.1%)	235 (53.9%)	0.006
	Ultimately fatal diseases	39 (35.8%)	141 (32.3%)	
	Rapidly fatal diseases	13 (11.9%)	38 (8.7%)	
	Unknown	10 (9.2%)	22 (5.0%)	
**ASA (American Society of Anesthesiology) classification**	Normally health patient	32 (29.4%)	146 (33.5%)	0.494
	Patient with mild systemic diseases	27 (24.8%)	85 (19.5%)	
	Patient with severe systemic disease that is not incapacitating	32 (29.4%)	100 (22.9%)	
	Patient with incapacitating systemic diseases that is a constant threat to life	14 (12.8%)	65 (14.9%)	
	Unknown	4 (3.7%)	40 (9.2%)	0.021
**Central Vascular catheter**	Yes	5 (4.6%)	4 (0.9%)	
	No	104 (95.4%)	432(99.1%)	
**Peripheral vascular catheter**	Yes	83 (76.1%)	291(66.7%)	0.05
	No	26 (23.9%)	145(33.3%)	
**Urinary catheter**	Yes	33 (30.3%)	75 (17.2%)	0.003
	No	76 (69.7%)	361(82.8%)	
**Intubation**	Yes	18 (16.5%)	29 (6.7%)	
	No	91 (83.5%)	407(93.3%)	0.001
**Surgery since admission**	Yes	64 (58.7%)	146(33.5%)	0.000
	No	45 (41.3%)	290(66.5%)	
**Length of stay categorized**	<8 days (below the median	63 (57.8%)	209(47.9%)	0.06
	> = 8 days (above or equal to median)	46 (42.2%)	227(52.1%)	
**The patient received Antimicrobial**	Yes	104 (95.4%)	294(67.4%)	0.000
	No	5 (4.6%)	142(32.6%)	

Availability of waste management material, immune status of the patient, central vascular catheter, surgery for admission and the patient received antimicrobial at the time of the survey were the predictors of hospital acquired infection. Patients in wards with the presence of medical waste container in the room were 82% less likely to develop hospital acquired infection compared to the patients in the wards without medical waste container with, AOR 0.18: and 95% CI, (0. 03–0.98). The odds of developing hospital-acquired infection among patients with immune deficiency patients were 2.34 times higher compared to their counterpart patients with 95% CI: (1.17–4.69). The central vascular catheter was a risk factor for hospital-acquired infection with AOR 6.92 and 95% CI: 1.28–37.47. The odds of developing hospital-acquired infection among patients who had surgery since admission in the ward was 2.35 times higher compared with patients without surgery since admission in the ward with 95% CI:1.08–5.09. Antimicrobial use was also a risk factor for hospital-acquired infection with AOR of 8.63with 95% CI: (3. 11–23.95) (Table 2).

**Table 2:** Risk factors for HAI in teaching hospitals in Amhara region, Ethiopia 2015.

Characteristics		Cases	Controls	Crude OR (Odds Ratio) (95% CI)	Adjusted OR (Odds Ratio) (95% CI)
**Available hand washing material in ward**	Yes	45(41.3%)	238(54.6%)	0.38(0. 21–0.68) **	0.81(0.35–1.86)
	No	64(58.7%)	198(45.4%)	1	1
**Presence of medical waste container at room**	Yes	102(93.6%)	431(98.9%)	0.12(0.003-.03) **	0.18(0.03–0.97)
	No	7(6.4%)	5(1.1%)	1	1
**Sex**	Male	64(58.7%)	201((46.1%)	1	1
	Female	45(41.2%)	235(53.9%)	0.57(0.37–0.89) **	0.65(0.37–1.13)
**Immune deficiency**	No	55(50.5%)	279(64.0%)	1	1
	Yes	31(28.4%)	92(21.1%)	1. 78(1.03–3.04) *	2.34(1.17–4.69) **
	Unknown	23(21.1%)	65(14.9%)	1.86(1.03–3.37)*	1.26(0.61–2.59))
**McCabe score**	Non-Fatal diseases	47 (43.1%)	235(53.9%)	1	1
	Ultimately fatal diseases	39 (35.8%)	141(32.3%)	1.48(0.88–2.48)	1.34(0.63–2.85))
	Rapidly fatal diseases	13 (11.9%)	38 (8.7%)	1.76(0.86–3.60) *	2.51(0.84–7.44))
	Unknown	10 (9.2%)	22 (5.0%)	2.54(1.05–6.13) *	1.04(0.30–3.58))
**ASA classification**	Normally health patient	32 (29.4%)	146(33.5%)	1	1
	Patient with mild systemic diseases	27 (24.8%)	85 (19.5%)	1.68(0.91–3.13)	0.78(0.36–1.72)
	Patient with severe systemic disease that is not incapacitating	32 (29.4%)	100(22.9%)	1.84(0.98–3.47)	1.08(0.41–2.87)
	Patient with incapacitating systemic diseases that is a constant threat to life	14 (12.8%)	65 (14.9%)	1.20(0.55–2.59)	1.19(0.35–4.11)
	Unknown	4 (3.7%)	40 (9.2%)	0.28(0.07–1.03)	0.09(0.01–0.64) *
**Central Vascular catheter**	Yes	5 (4.6%)	4 (0.9%)	5.00(1.34–18.61) *	6.92(1.28–37.47) *
	No	104(95.4%)	432(99.1%)	1	1
**Peripheral vascular catheter**	Yes	83 (76.1%)	291(66.7%)	1.61(0.99–2.63)	1.17(0.59–2.32)
	No	26 (23.9%)	145(33.3%)	1	1
**Urinary catheter**	Yes	33 (30.3%)	75 (17.2%)	2.30(1.37–3.87) **	1.23(0.59–2.55)
	No	76 (69.7%)	361(82.8%)	1	1
**Intubation**	Yes	18 (16.5%)	29 (6.7%)	3.40(1.66–6.97) **	0.80(0.28–2.31)
	No	91 (83.5%)	407(93.3%)	1	1
**Surgery since admission**	Yes	64 (58.7%)	146(33.5%)	3.31(2.05–5.36) **	2.35(1.08–5.09) *
	No	45 (41.3%)	290(66.5%)	1	1
**Patient received Antimicrobial**	Yes	104(95.4%)	294(67.4%)	10.69(4.22–27.07) **	8.63(3.11–23.95) ***
	No	5 (4.6%)	142(32.6%)	1	1

* Statistically significant association P<0.05

**strong statistically significant p between 0.001 & 0.05

*** Very strong, statistically significant P<0.001.

## Discussion

Risk factors for hospital-acquired infection are dynamic and complex phenomena in the hospital. In this matched case control study, availability of waste management material in the room, immune status of the patient, central vascular catheter, surgery since admission and patient received antimicrobial at the time of survey were the predictors of hospital acquired infection.

Most of the studies on risk factors for hospital-acquired infection employed by cross-sectional methods, that has a limitation of statistical power by comparing the groups internally. The introduction of bias in matched case control study is lower than in the cross-sectional design [35]. Matched case control study design is assumed to generate valid data impacting the reduction of bias among main exposure variables.

The availability of hand-washing facilities waste management material in the ward are important to prevent hospital acquired infection[37]. The presence of alcohol- based hand rub material in the ward significantly increases the consumption of alcohol-hand rub material in the ward[38]. Thus availability of hygiene maintenance materials and behaviors of healthcare workers reduced healthcare associated infections[39–41]. In Ethiopia researches showed low level of hand hygiene practice[42] and poor condition of medical waste management in health care facilities [43]. The availability of waste management materials is believed to reduces the exposure of waste to patients and health care workers.

This study found immune deficiency of patients as a risk factor for hospital acquired infection. This finding was also supported by another finding, in which patients with high compromised health status were at risk for hospital acquired infection[44].

In the modern healthcare system, the live saving invasive treatment devices and procedures are found to increasingly threaten patients. For example, while catheters provide lifesaving therapy, they also have an iatrogenic effect, by being a route of transmission of microorganisms to the patient’s body, thereby causing infection. Hospital-acquired infections occur with the devices used in medical procedures[45]. A hospital based research in Poland showed a positive correlation between prevalence of hospital-acquired infection and exposure to invasive procedures[22]. In our study, central vascular catheter used on patients was 7.56 times more likely to be a risk factor compared to non-central vascular catheter patients. This finding was supported by other studies conducted in hospitals in Poland, Morocco and China [22,26,46].

The high risk of central vascular catheter may be due to low level of implementation of a multidimensional infection control strategy in the study setting. Implementation of Nosocomial Infection Control Consortium (INICC) bundles and other infection control measures in some of the developing countries reduced devise-associated infection of CLABSI[47–50], VAP[49,51,52] and CAUTI [53] in ICU patients. Surgery since admission was one of the determining factors for hospital-acquired infection in this finding. This was also supported by other findings of pilot point prevalence study conducted by the European Centre for Disease Prevention and Control (ECDC) [54] and China and Poland[23,26].

A research showed that, antimicrobial prescribing with broad-spectrum antibiotics to patients increased the risk of hospital acquired Drug-Resistant *Acinetobacter baumannii* infections[44]. A similar finding was also observed in Morocco that; antimicrobial use was associated with hospital-acquired infections[46]. Our study supported the above finding in which a patient received antimicrobial was 9.16 times more likely to acquire hospital-acquired infections compared to those who didn’t take antimicrobials.

This study has limitations. In this research, we focused on a relatively small number of risk factors for hospital-acquired infections. Some of the clinical data in the hospital recording system was incomplete. This may introduce bias in statistical analysis. Evidence for this finding can be generalized to similar resource limited settings. Because, the data were collected from a large proportion of controls to cases, it gives high power to identify the variations in the population. The other limitation in this study was that controls did not match by type of ward and length of stay with cases. This may minimize the power of the study to identify confounders in the study.

## Conclusions

Despite the above limitations, the risk factors identified in this study are very important for the prevention and control of hospital-acquired infection in teaching hospitals in Ethiopia. Presence of medical waste container in the room, patients’ immune status, central vascular catheter, surgery since admission and patients received antimicrobials were the independent predictors for hospital acquired infections. The managers and medical workforce should consider the availability of healthcare facilities. Hospitals and clinicians need to follow the appropriate safe medical procedures for use of external devices and give attention to the immunocompromised patients for the prevention and control of hospital-acquired infections.

## Supporting information

S1 Supporting Information (ZIP)
